# Coiled‐coils: The long and short of it

**DOI:** 10.1002/bies.201600062

**Published:** 2016-08-05

**Authors:** Linda Truebestein, Thomas A. Leonard

**Affiliations:** ^1^ Department of Structural and Computational BiologyMax F. Perutz Laboratories (MFPL)Vienna Biocenter (VBC)ViennaAustria; ^2^ Department of Medical BiochemistryMedical University of ViennaViennaAustria

**Keywords:** allostery, coiled‐coil, molecular ruler, molecular spacer, scaffold

## Abstract

Coiled‐coils are found in proteins throughout all three kingdoms of life. Coiled‐coil domains of some proteins are almost invariant in sequence and length, betraying a structural and functional role for amino acids along the entire length of the coiled‐coil. Other coiled‐coils are divergent in sequence, but conserved in length, thereby functioning as molecular spacers. In this capacity, coiled‐coil proteins influence the architecture of organelles such as centrioles and the Golgi, as well as permit the tethering of transport vesicles. Specialized coiled‐coils, such as those found in motor proteins, are capable of propagating conformational changes along their length that regulate cargo binding and motor processivity. Coiled‐coil domains have also been identified in enzymes, where they function as molecular rulers, positioning catalytic activities at fixed distances. Finally, while coiled‐coils have been extensively discussed for their potential to nucleate and scaffold large macromolecular complexes, structural evidence to substantiate this claim is relatively scarce.

## Introduction

A typical mammalian cell contains on the order of 10^10^ protein molecules at a concentration of 2–4 × 10^6^ molecules per cubic micrometer 1. That same cell contains hundreds of organelles, three billion bases of genetic code, and a vast array of sugars and small molecule metabolites. The cell must exchange material with its extracellular environment, respond to environmental cues, maintain, duplicate, and segregate its genetic material, and accomplish all of these tasks in a manner that benefits the entire organism. Not surprisingly, intracellular organization and compartmentalization are critical to its coordinated behavior. Within this framework, size matters: all of the parts must be positioned correctly and made to work in unison. Similarly, efficiency in signaling or metabolic pathways depends on the effective presentation of substrate and enzyme, while undesired cross talk depends on their effective insulation.

Here, we take a look at the importance of size at the molecular level: we examine the use of coiled‐coils as structural domains and their roles in protein function. We discuss examples of coiled‐coils as molecular spacers as well as coiled‐coils that communicate conformational changes along their length. We summarize recent examples of molecular rulers and the implications of confining catalytic activity to a fixed position. Finally, we discuss the role of coiled‐coils in scaffolding protein complexes.

## Coiled‐coils are structurally conserved

In 1952, Francis Crick described a new type of protein structure, which he called the coiled‐coil 2. Hypothetical at the time, the coiled‐coil was proposed as a solution to the X‐ray fiber diffraction patterns of α‐keratin, which showed a strong reflection at 5.1 Å compared to the reflection at 5.4 Å observed for a canonical α‐helix. Crick correctly predicted that by supercoiling an α‐helix, one could obtain a repeating structure in which two or more adjacent α‐helices could pack together in a knobs‐into‐holes arrangement. Such a structure is impossible to obtain from two straight helices arranged side by side due to the non‐integral nature of the helix. Crick proposed that the energy required to deform the helices could be compensated for by the closer packing of the side chains. The structure of influenza virus hemagglutinin 3 later confirmed the basic parameters of the coiled‐coil 4, 5. Indeed, Crick's parametric equations, which describe coiled‐coils using just four parameters, are able to reproduce the majority of subsequently experimentally determined coiled‐coil structures and are at the heart of CCCP and CC‐builder, two web‐based tools for designing, building, and assessing coiled‐coil assemblies 6, 7.

**Figure 1:**
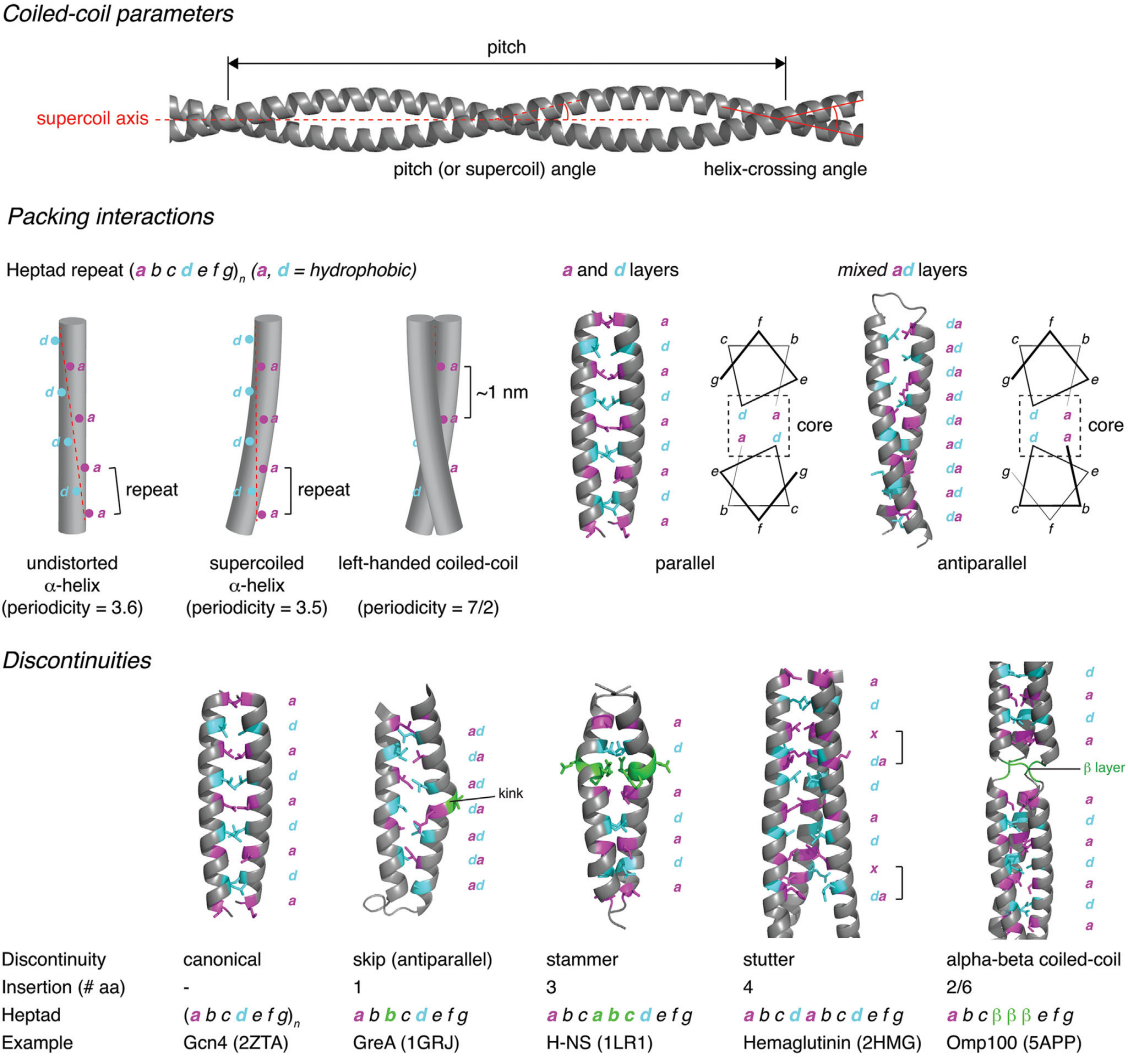
Coiled‐coil architecture. **Coiled‐coil parameters.** The basic parameters of the coiled‐coil are its pitch (periodicity of the supercoil), the associated pitch (or supercoil) angle (angle of the helix with respect to the supercoil axis), and the helix‐crossing angle (angle at which the two helices cross each other). **Packing interactions.** The canonical coiled‐coil is characterized by a heptad repeat in which hydrophobic residues are conserved at positions *a* and *d*. Undistorted α‐helices cannot pack together in a side‐by‐side arrangement due to the non‐integral periodicity of the helix (3.6). By supercoiling the helices, the periodicity is reduced to 3.5, leading to the 7/2 periodicity of a canonical, left‐handed coiled‐coil, with each heptad repeat measuring ∼1 nm along the coiled‐coil. Knobs‐into‐holes packing of two parallel, supercoiled helices results in *a* and *d* layers, while antiparallel helices exhibit mixed *ad* layers. **Discontinuities.** Insertions of 1, 2, 3, 4, or 6 amino acids give rise to discontinuities in the heptad repeat and local structural deformations in the coiled‐coil. Examples are shown for insertions of 1, 3, 4, and 2/6 amino acids in comparison to the canonical coiled‐coil of GCN4.

The canonical coiled‐coil is formed from a heptad repeat, labeled *abcdefg*, in which hydrophobic amino acids at positions *a* and *d* are conserved (Fig. 1). The conservation of hydrophobic residues alternately three and four residues apart (average 3.5) is close to the 3.6 amino acids per turn periodicity of a regular α‐helix. Consequently, helices deriving from such repeating sequences exhibit distinct amphipathic character, with both hydrophobic and polar faces. The association of two helices via their hydrophobic faces drives coiled‐coil formation. However, in order to pack two helices together and maintain hydrophobic contacts, the knobs‐into‐holes packing of side chains requires that these residues occupy equivalent positions, turn after turn. By supercoiling the helices around each other, the periodicity is effectively reduced from 3.6 to 3.5 with respect to the supercoil axis. This allows the positions of side chains to repeat after two turns, or seven residues, instead of drifting continuously on the helical surface 8.

Coiled‐coil domains have since been shown to be ubiquitous repetitive peptide motifs present in all the domains of life 9. In total, coiled‐coil forming sequences comprise up to 10% of an organism's proteome 9, 10, 11. Naturally occurring coiled‐coils comprise between two and six helices arranged either parallel or antiparallel to each other. They may be homo‐ or hetero‐oligomers, and may be formed from separate chains, or from consecutive helices of the same chain. While variations on the canonical heptad repeat are known, these non‐canonical motifs are also based on repeating hydrophobic and polar residues spaced three or four residues apart. These coiled‐coils exhibit slight variations in the knobs‐into‐holes packing and different degrees of supercoiling 8, 12. Discontinuities caused by the insertion of one, three, or four residues result in local deformations in the coiled‐coil assembly (Fig. 1), while insertions of two or six residues strain the supercoil to breaking point, leading to the local formation of β‐strands that move the path of the chain by 120° around the trimer axis. The resulting structure is a so‐called α‐β coiled‐coil, in which the β‐strands associate to form a triangular structure called a β‐layer (Fig. 1). Hartmann and co‐woorkers show that β‐layers are found in naturally occurring proteins in which they form fibers with repeating α and β structure 13. Clear sequence to structure relationships 14 coupled with their intrinsic properties of periodically repeating structure, capacity for self‐assembly, and mechanical strength have seen coiled‐coils feature prominently in recent protein engineering efforts 15, 16, 17, 18. While the applications for de novo coiled‐coil design and prospects for synthetic biology fall outside the scope of this review, the potential to inform our understanding of coiled‐coil structure, folding, and function as well as for innovation in biotechnology, material science, and medicine is manifold 19.

Based on the underlying sequence repeats that govern their assembly, coiled‐coils can be reliably predicted from primary sequence 20. However, a more recent extension of the SUPERFAMILY database for coiled‐coil domains has improved these predictions by incorporating existing genomic and structural information 11. Examples of structurally validated coiled‐coils of different architectures may be found in the periodic table of coiled‐coil structures 21, while the CC+ database 22 of coiled‐coil structures (http://coiledcoils.chm.bris.ac.uk/ccplus/search/dynamic_interface) is a useful resource for structural and cell biologists alike. The rest of this review will focus on the biological roles of coiled‐coil domains.

## Coiled‐coils function as molecular spacers

While at least one coiled‐coil has been shown to possess catalytic activity 23 and the coiled‐coil is widely used to facilitate oligomerization, many coiled‐coils have been proposed to act as molecular spacers that either separate functional domains or scaffold large macromolecular complexes. As such, their physical properties, especially their length and flexibility, have important structural and functional consequences. Bioinformatic analysis of coiled‐coil regions has shown that length variation of coiled‐coil sequences is, on average, 3.6 times lower than in non coiled‐coil regions 24. While both length and primary sequence are, on average, well conserved, length conservation is not correlated with sequence conservation. As such, other selective pressures must account for length conservation when sequences diverge, implying that the physical size of the domain is a critical factor in its biological function.

Early studies in the 1990s identified a number of coiled‐coil proteins with apparent roles as molecular spacers. Omp‐α, an outer membrane protein in *Thermotoga maritima* with a predicted coiled‐coil domain, was observed to span the periplasmic space 25. Electron micrographs revealed Omp‐α to be a rod‐like particle 50 nm in length, corresponding to the distance between the inner and outer membranes. The rod domain of Omp‐α has since been modeled as a four‐stranded coiled‐coil 26, though this remains to be confirmed with a high‐resolution structure. Around the same time, a structural protein of the yeast spindle pole body, Spc110 (Nuf1p), was characterized. Spc110 links the inner and central plaques of the spindle pole body with a rod‐like coiled‐coil, 73 nm in length. Truncations in the coiled‐coil domain gave rise to predictable changes in the structure of the spindle pole body, indicating the significance of the spacer length 27.

## Coiled‐coil proteins mediate vesicle tethering

The longest coiled‐coil identified to date, found in the protein Giantin, is remarkably well conserved in length, but not sequence, in vertebrates (Table 1). Since its discovery, it has been suggested to form inter‐membrane cross‐bridges in the Golgi 28 and, more recently, to tether COPI vesicles to the Golgi 29, 30, 31. The coiled‐coil domain functions as a spacer of approximately 0.5 µm between a short non‐coil sequence at its N‐terminus that binds the Golgi and a C‐terminal transmembrane segment that captures vesicles. An analysis of coiled‐coil domain‐containing proteins in which the coiled‐coil is believed to act as a molecular spacer concluded that these coiled‐coils exhibit maximal sequence divergence 32.

**Table 1:** Length and sequence conservation in coiled‐coil proteins

Protein	*H. sapiens* coiled‐coil segment (no. of amino acids)	*D. rerio* coiled‐coil segment (no. of amino acids)	Sequence conservation (coiled‐coil/whole)	Length conservation (*H. sapiens*/*D. rerio*)
Golgins				
Giantin	48–3103 (3056)	49–3286 (3238)	33.5/33.9	3259/3356
Centriole				
Sas‐6	161–484 (324)	155–486 (332)	61.6/57.4	657/627
NuMA	213–1699 (1487)	212–1985 (1774)	29.4/31.3	2115/2446
Kinetochore				
Ndc80	261–403 (143)	263–419 (157)	49.5/44.1	642/632
	458–642 (185)	459–632 (174)	33.5/44.1	
SMC proteins				
SMC1	187–511 (325)	187–511 (325)	84.9/89.2	1233/1232
	656–1067 (412)	656–1067 (412)	87.6/89.2	
SMC2	177–507 (331)	177–507 (331)	69.5/74.4	1197/1199
	670–1033 (364)	670–1033 (364)	68.1/74.4	
SMC3	170–503 (334)	170–503 (334)	94.9/95.5	1217/1216
	672–1009 (338)	672–1009 (338)	94.1/95.5	
SMC4	283–591 (309)	284–591 (308)	59.0/68.6	1288/1289
	767–1020 (254)	768–1029 (243)	55.6/68.6	
SMC5	207–459 (253)	262–447 (185)	44.1/57.0	1101/1073
	648–930 (283)	636–903 (268)	45.7/57.0	
SMC6	226–451 (226)	276–488 (213)	31.1/45.6	1091/1093
	660–910 (251)	681–939 (259)	34.6/45.6	
Rad50	222–1076 (855)	242–1096 (855)	63.6/71.0	1312/1312
Dynein‐1	3189–3275 (87)	3187–3275 (89)	95.4/91.0	4646/4643
	3396–3500 (105)	3396–3499 (104)	97.1/91.0	
Kinesins				
kinesin‐1	330–914 (585)	330–917 (588)	84.6/86.4	969/963
kinesin‐5	364–480 (117)	369–480 (112)	56.4/51.1	1056/955
Myosin‐2	845–1941 (1097)	841–1937 (1097)	83.0/82.6	1941/1937
Kinases				
ROCK1	407–1120 (714)	409–1125 (717)	65.4/75.5	1354/1359
ROCK2	414–1143 (730)	409–1142 (734)	68.9/75.9	1379/1375
MRCKα	438–943 (506)	434–943 (510)	64.0/73.7	1732/1716
MRCKβ	422–939 (518)	433–939 (507)	74.0/74.0	1711/1708
DMPK	457–535 (79)	473–523 (51)	34.7/53.0	629/600

Coiled‐coil domains were predicted for selected proteins in both humans and zebrafish using COILS 115. Zebrafish encodes genes for all the selected proteins, but the vertebrate lineages of humans and zebrafish diverged more than 400 m years ago. Sequence conservation is presented for both the predicted coiled‐coil and the whole protein. To avoid errors in the length of the coiled‐coil domains that arise from the prediction algorithm, the length conservation is presented only for the full‐length proteins.

Several other long‐coiled‐coil proteins are found on the Golgi, where they are typically anchored to the membrane via a transmembrane domain or by binding to a small GTPase (reviewed in 33). Collectively referred to as Golgins, these proteins form parallel homodimeric coiled‐coils over most of their length, extending ∼50–600 nm from both the cis‐ and trans‐faces of the Golgi. Many Golgins have binding sites for Rab family GTPases along their length, suggesting a role in vesicular trafficking though it should be noted that structural evidence for the interaction of Golgins with small GTPases is still missing. Two major studies support their role in vivo as tethers. Using purified Golgins and isolated vesicle fractions, Malsam et al. showed that different Golgins could capture COPI vesicles with specificity for certain sub‐populations of vesicles 34. More recently, Wong and Munro investigated the ability of Golgins to transport vesicles in cells, demonstrating that individual Golgins were sufficient to tether distinct classes of transport vesicles 35. Interestingly, the coiled‐coil regions of Golgins are characterized by numerous short breaks, which likely confer conformational plasticity 36, a property that may underlie their capacity to mediate vesicle tethering. A recent study of the Golgin GCC185 found that GCC185 is shorter than predicted and that its N‐ and C‐termini are within 40 nm of each other on the Golgi 37. This flexibility was suggested to arise from the local unwinding of a central region of the coiled‐coil, allowing GCC185 to capture vesicles and bring them into close proximity with the Golgi membrane.

## Coiled‐coil proteins are essential for accurate chromosome segregation

**Figure 2:**
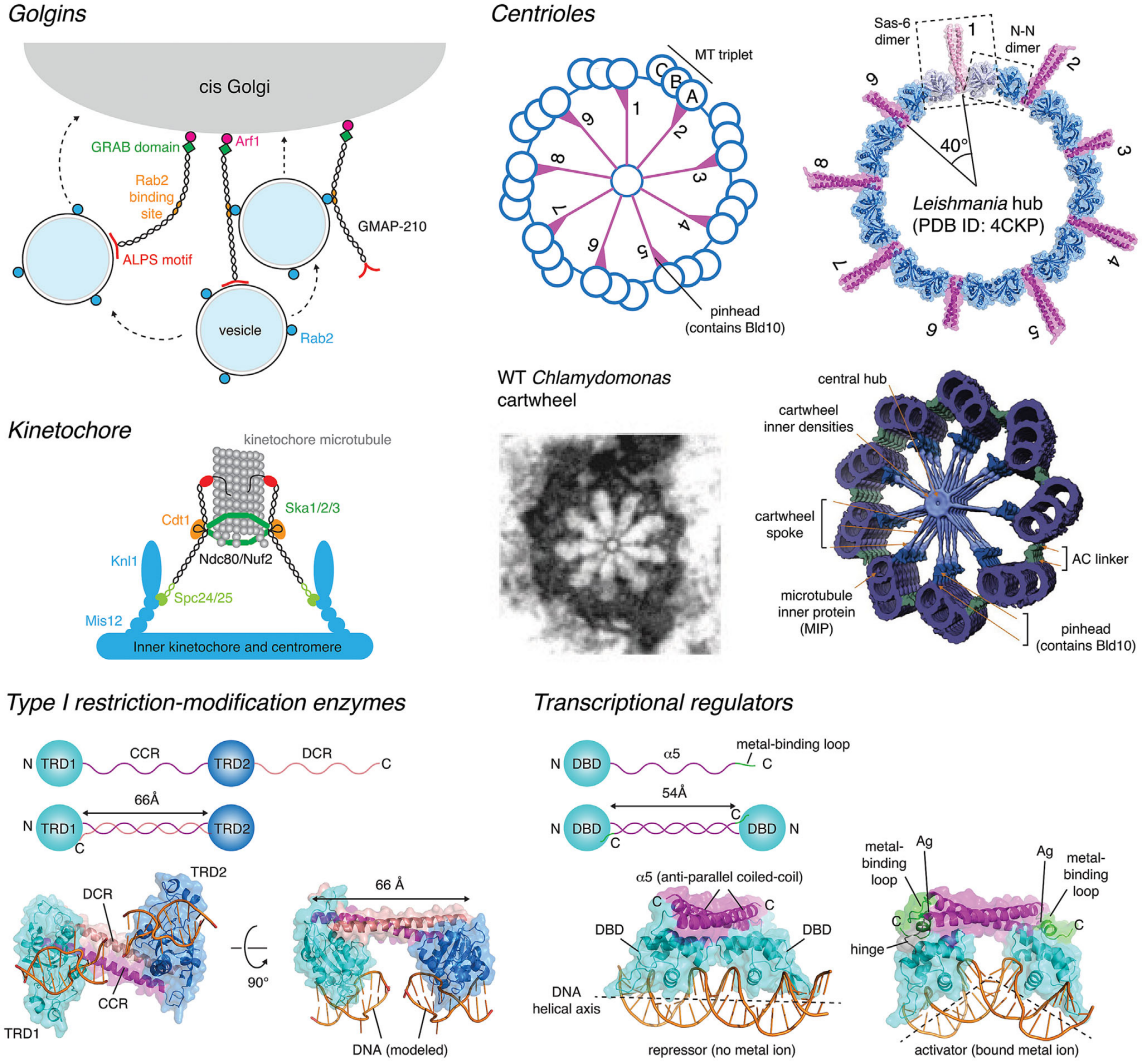
Coiled‐coils used as molecular spacers. **Golgins.** The Golgins are a family of tethering proteins with long coiled‐coils that capture vesicles on the surface of the Golgi. GMAP‐210 contains a curvature‐sensing ALPS motif in its C‐terminus, which allows it to selectively capture and tether vesicles in vitro and in vivo 116, 117, 118. This panel is based on Fig. 2 from Witkos and Lowe 33. **Kinetochore.** Ndc80 is a coiled‐coil component of the kinetochore that, in combination with the coiled‐coil proteins Spc24 and Spc25, links the centromere to the spindle microtubule. Ndc80 is highly conserved in length (Table 1). The Ska complex (Ska1/2/3) of coiled‐coil proteins forms a W‐shaped dimer of 35 nm that binds to kinetochore microtubules and is essential for mitosis. This panel is based on Fig. 1 from Matson and Stukenberg 119. **Centrioles.** Sas‐6 forms a coiled‐coil dimer. Dimerization of the head domains of Sas‐6 with neighboring Sas‐6 molecules leads to formation of the centriolar hub, a cartwheel with ninefold symmetry in which the coiled‐coil domains of Sas‐6 form the spokes. Bld10p, another coiled‐coil protein essential for centriole assembly, stabilizes the ninefold symmetric arrangement of the centriole via a yet to be determined mechanism. The ninefold symmetric cartwheel was recently visualized in a crystal structure of *Leishmania* Sas‐6 50, reflecting the structure of centrioles visualized previously by electron microscopy 51 and cryo‐electron tomography 53, 54 (reproduced with permission). **Type I restriction‐modification enzymes.** The DNA binding domains are held at a fixed distance apart by an intramolecular anti‐parallel coiled‐coil. The arrangement permits the recognition of two DNA sequences separated by a fixed spacer, and recruits a restriction enzyme that cuts randomly either side 55. **Transcriptional regulators.** Dimerization of CueR via an antiparallel coiled‐coil maintains the DNA binding domains (DBD) at a fixed distance of 55 Å that permits the recognition of successive major grooves of B‐DNA via a recognition helix in the DBD. Binding of heavy metals to a metal‐binding loop at the C‐terminus of CueR results in an allosteric change in the conformation of the DBDs with respect to the coiled‐coil. This conformational change distorts B‐DNA to a more open A‐DNA, converting CueR from a transcriptional repressor into an activator 57.

Coiled‐coil proteins that function as molecular spacers are also found in the kinetochore. Kinetochores are protein structures that link chromatids to the mitotic spindle during cell division, contraction of which segregates the replicated DNA into the two daughter cells. Ndc80 is a coiled‐coil protein spanning the inner and outer kinetochore, invariant in length in vertebrates (Table 1). Together with the coiled‐coil domain‐containing proteins Nuf2p, Spc24p, and Spc25p, it forms a heterotetrameric complex, which resembles a rod of approximately 57 nm with globular domains at either end 38. The Ndc80 complex links the centromere to microtubule structures in the trilaminar kinetochore 39, 40 (Fig. 2). Ndc80 also recruits the spindle assembly checkpoint (SAC) kinase Mps1 to the outer kinetochore where it phosphorylates Spc105, leading to cell cycle arrest during metaphase. While Ndc80 has been suggested to function as a mechanical switch that regulates the SAC by physically separating Mps1 (kinase) and Spc105 (substrate) in response to end‐on microtubule attachment 41, Mps1 has also been shown to be competitively displaced from the kinetochore by microtubules themselves 42, 43. In addition to Ndc80, the Ska complex is essential for accurate cell division. Ska1, Ska2, and Ska3 form a 35 nm W‐shaped dimer of intertwined coiled‐coils that binds microtubules and is essential for mitosis. Mutations in the dimer interface lead to chromosome congression failure and eventual cell death 44. Indeed, many other kinetochore proteins are predicted to contain coiled‐coil domains, and disruption of the network of coiled‐coil interactions leads to defects in kinetochore assembly and cell division 45.

Essential for accurate positioning and elongation of the mitotic spindle, the nuclear mitotic apparatus (NuMA) protein contains a 1500 amino acid coiled‐coil separating globular N‐ and C‐terminal domains which bridge the minus end‐directed microtubule motor protein complex dynein to the plasma membrane during metaphase 46. In addition to recruitment of dynein, the microtubule‐binding domain of NuMA, which binds to the tips of the microtubules comprising the mitotic spindle, is essential for NuMA's ability to position the spindle accurately 47. NuMA is almost invariant in length among vertebrates, despite enormous sequence divergence (Table 1), suggesting that the length of its coiled‐coil is critical for its function.

## Coiled‐coil proteins control centriole architecture

Centrioles are intracellular organelles found in most eukaryotic cells and an important component of centrosomes in the metazoan lineage, where they act as the microtubule organizing center (MTOC) of the cell. Centrosomes are essential for organizing the mitotic spindle during cytokinesis. In organisms with flagella and cilia, the mother centriole becomes the basal body of the cilium or flagellum. Centrioles are, therefore, critical regulators of ciliogenesis and the associated capacities for locomotion, sensation, and signaling in these organisms. Centrosomes are abundant in coiled‐coil proteins, the evolution of which led to the formation of a signaling hub that scaffolds ancestral eukaryotic proteins crucial for multicellular development 48. Two key components of the central hub of centriole assembly in *Caenorhabditis elegans* are Sas‐6 and Bld10p. Sas‐6 comprises an N‐terminal coiled‐coil domain and C‐terminal dimerization domain that cooperate to form a ninefold symmetric assembly of radiating spokes 49, 50, the length of which is conserved among vertebrates (Table 1, Fig. 2). Bld10p is a 1600 amino acid coiled‐coil protein that stabilizes the 9‐fold symmetric arrangement of the spokes. Truncations of Bld10p in *Chlamydomonas* give rise to shorter spoke lengths and increased frequencies of centrioles with lower symmetry 51, while *Tetrahymena* missing Bld10 show microtubule misorientation and basal body disassembly 52. Sas6 and Bld10p, therefore, appear to be molecular spacers whose lengths dictate the evolutionarily conserved ninefold symmetric centriolar structure. Cryo‐electron tomography studies have revealed important details of how the cartwheel hub is connected to the microtubule triplets 53, 54, including the region containing Bld10p, but further studies will be required to determine precisely how Bld10p stabilizes the ninefold symmetric arrangement.

## Coiled‐coils control DNA recognition and cleavage

The type I restriction‐modification enzymes are another elegant example of the use of a coiled‐coil molecular spacer to guide catalytic activity. Type I restriction‐modification enzymes recognize two specific dsDNA sequences separated by a spacer of definite length, but cleave randomly away from the recognition sequences. They comprise specificity, methylation, and restriction subunits. The crystal structure of the specificity subunit of *Methanococcus jannaschii* provides a molecular explanation for how two sequences a fixed distance apart are recognized 55. The specificity subunit encodes two DNA‐binding domains separated by a central coiled‐coil region (CCR) and a second conserved coiled‐coil at the distal C‐terminus (DCR) (Fig. 2). The CCR and DCR together form an antiparallel coiled‐coil 66 Å in length, separating the two DNA‐binding domains. The coiled‐coil serves as a molecular ruler to guide the recognition of tandem sequence motifs. Catalytic activity of the restriction subunit is, therefore, restricted to sequences surrounding the recognition sequences.

A similar structural organization of DNA‐binding domains can be observed in the MerR family of bacterial transcriptional activators 56. While the DNA‐binding domains are unrelated to those of the type I restriction‐modification enzymes, dimerization of their C‐terminal α‐helices to form an antiparallel coiled‐coil maintains the two domains at a fixed distance with respect to each other (Fig. 2). Given the structural parameters of DNA, it is not coincidental that the coiled‐coil is similar in length to that of the type I restriction‐modification enzymes. The recent determination of the structure of CueR, a metallo‐sensor transcriptional regulator, in complex with its cognate promoter, illustrates how the binding of metal ions to an allosteric site switches CueR from a repressor into an activator 57. In both the repressor and activator conformations, the coiled‐coil of CueR maintains the two DNA‐binding domains at the correct distance apart to recognize successive major grooves of DNA.

## Beyond simply a molecular spacer – where sequence matters

**Figure 3:**
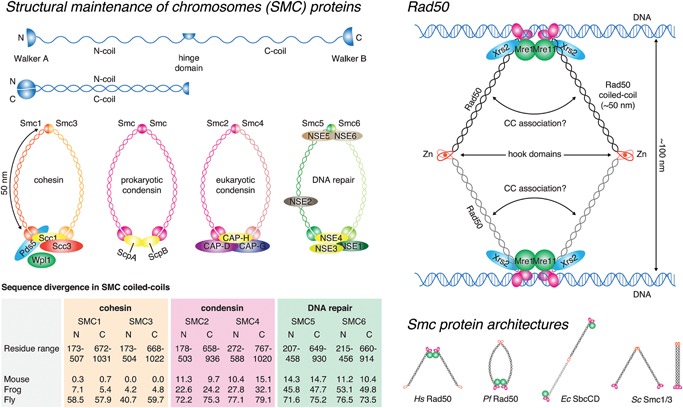
SMC proteins – beyond simply a molecular spacer. **Structural maintenance of chromosomes (SMC) proteins.** SMC proteins are characterized by N‐ and C‐terminal Walker ATPase domains, separated by two stretches of sequence that fold back upon each other to form an antiparallel coiled‐coil. A hinge domain in the middle mediates dimerization of two SMC proteins in cohesin, condensin, and the DNA repair complex of SMC5/6. The SMC proteins are conserved in both length and sequence. Eukaryotic cohesin (SMC1/3), in particular, exhibits almost invariant sequence in vertebrates. This panel was adapted from Box 1 of Jeppsson et al. 120 with permission. **Rad50.** Closely related to the SMC proteins is the DNA damage response regulator Rad50, which contains an antiparallel coiled‐coil of 50 nm, the same length as SMCs. Rad50 dimerizes via a specialized zinc‐hook domain, the Rad50 counterpart of the SMC hinge domain, allowing it to bridge sister chromatids during meiotic recombination. Evolutionarily conserved in length, truncations in the coiled‐coil lead to defects in Rad50 function 60. This panel is based on Fig. 1a of Hohl et al. 60. **Smc protein architectures.** A number of Smc/Rad50 structures have been observed in vitro from the closed circle (mediated by dimerization of the hinge and Walker ATPase domains) to linear dimers and rod‐shaped dimers (in which the coiled‐coils pack together side‐by‐side).

While the Golgins and other coiled‐coils exhibit high levels of sequence substitution, the coiled‐coils of the cohesin subunits are the most conserved naturally occurring coiled‐coils (Table 1, Fig. 3). Cohesins are members of the structural maintenance of chromosomes (SMC) family of proteins, central regulators of chromosome structure in all branches of life. Comprising N‐ and C‐terminal Walker ATPase domains and a central hinge domain separated by two α‐helical segments, the SMC proteins form an intramolecular antiparallel coiled‐coil, approximately 50 nm in length (Fig. 3). Six eukaryotic SM proteins pair to form three discrete complexes (cohesin, condensin, and SMC5/6) with essential functions in DNA replication, maintenance, and repair. Beyond the conservation of the length of their coiled‐coils, the extreme level of sequence conservation in cohesin (SMC1/3) implies that almost the entire surface of the coiled‐coil is essential for sister chromatid cohesion. A recent structural study of SMC proteins showed that the coiled‐coil domains of prokaryotic Smc‐ScpAB and eukaryotic condensin associate in solution to form a closed rod‐like conformation in which the SMC coiled‐coils are closely juxtaposed along their length. DNA‐binding to the hinge causes opening of the SMC arms, leading to the proposal that the head domains transmit an ATP‐dependent conformational change via the coiled‐coil to expose a DNA‐binding site on the inner surface of the hinge domain 58. This structural transition between closed and open conformations is believed to underlie the loading of DNA into cohesin rings 59.

Rad50 is a central regulator in the DNA damage response pathway. Like the SMCs, Rad50 comprises a long internal coiled‐coil that folds back upon itself to bring globular N‐ and C‐terminal domains together to form a functional ABC ATPase domain (Fig. 3). The hinge region of the coiled‐coil contains a zinc hook motif that permits dimerization of the protein. Consistent with the high conservation of both coiled‐coil length and sequence (Table 1), truncations of the Rad50 coiled‐coil abolish telomere maintenance and meiotic double strand break (DSB) formation, and severely impair homologous recombination 60. While it is clear that long‐range interactions are essential for Rad50 function, it is also clear that the coiled‐coil domain is a mechanical transducer of conformational changes in the globular domains 61, 62, 63, 64, 65, 66 and that truncations likely affect not just length, but also the disposition of the globular domains. How the SMCs, and indeed other coiled‐coils, such as myosin, transmit signals along the length of their structure remains an outstanding question.

The coiled‐coil tail domain of myosin, highly conserved in both length and sequence (Table 1), self assembles in an anti‐parallel fashion to form the bipolar thick filament. While the contraction of skeletal muscle is widely attributed to exclusive regulation of the actin‐based thin filaments, a recent study has revealed regulation of the thick filament under high load 67. The authors propose that the increase in periodicity of the thick filament under high isometric tension results in the release of myosin motors from their inhibited conformation on the thick filament, thereby allowing further force generation. While the mechanisms of thick filament lengthening and motor domain exposure are not known, these results illustrate the potential of the coiled‐coil domains of the thick filament to act as mechanosensors and to transmit structural changes along their length.

## Coiled‐coil domains communicate conformational change

Allosteric communication along coiled‐coils requires concerted changes in the register and packing of the chains. The capacity to transmit structural changes along the length of a coiled‐coil has recently been shown to be essential for the activity of motor protein complexes involved in the cellular transport of cargo.

**Figure 4:**
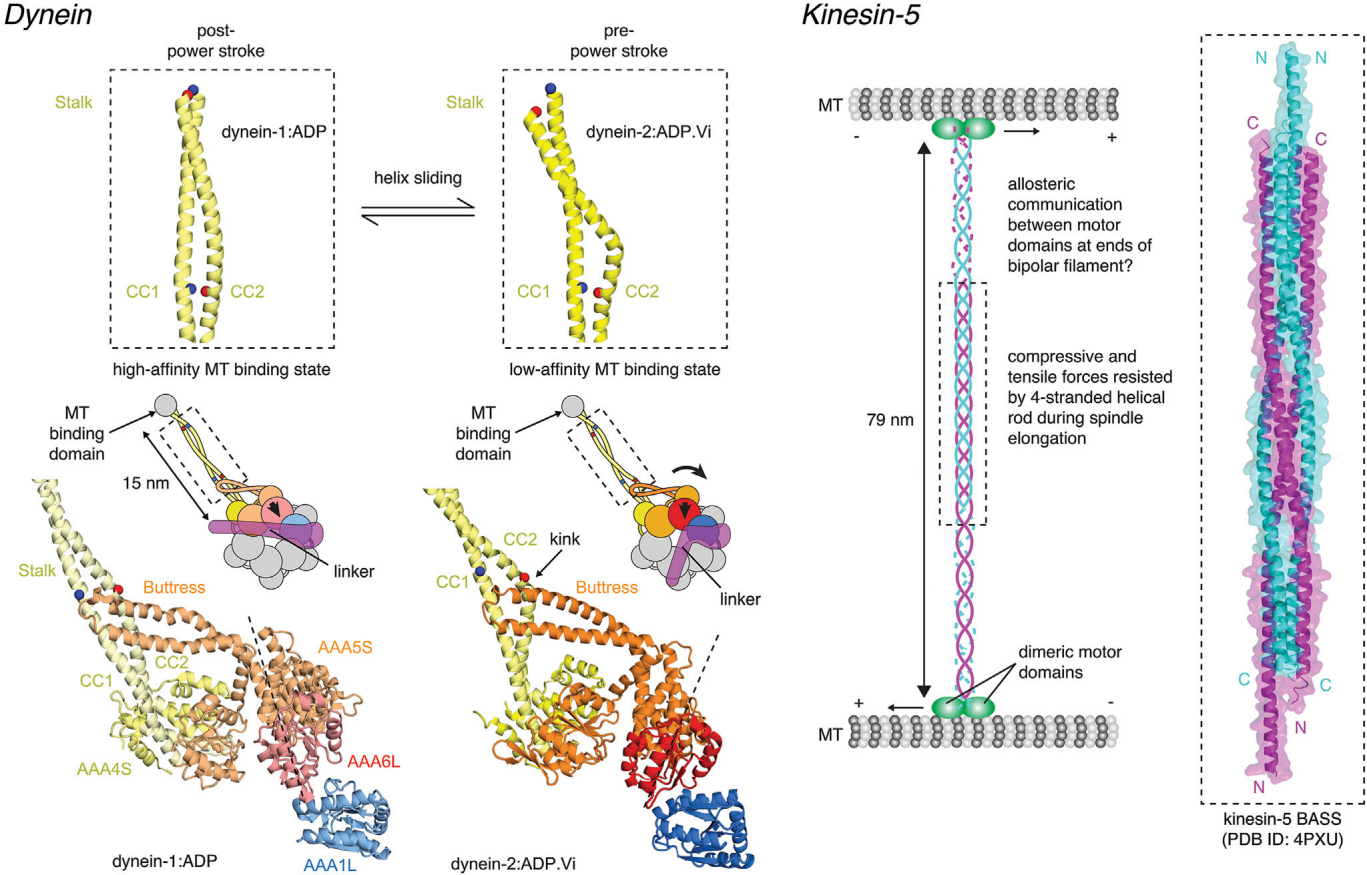
Allosteric communication by coiled‐coils. **Dynein.** Dyneins are minus‐end directed motors highly conserved in both length and sequence (Table 1). The hexameric AAA ATPase motor domains are bridged to the microtubule‐binding domain by a coiled‐coil stalk, 15 nm in length. Conformational changes in the motor domain upon ATP hydrolysis in AAA1 are propagated to the stalk via the buttress. The buttress pulls on one helix of the coiled‐coil in the stalk, causing it to shift register by one full turn of the α‐helix. The sliding of the helices with respect to one another causes, in turn, a conformational change in the microtubule‐binding domain at the tip of the stalk, driving it into a low‐affinity conformation and promoting microtubule release. Microtubule re‐binding leads to a force‐producing swing of the linker (power stroke) back to its preferred straight conformation (post‐power stroke) and the release of ATP hydrolysis products resets the cycle. This figure was adapted from Fig. 4 of Schmidt et al. 69 with permission. **Kinesin‐5.** Kinesin‐5 is a homotetrameric bipolar plus‐end directed motor essential for spindle assembly and elongation during anaphase. Kinesin‐5, like kinesin‐1, is highly conserved in length (Table 1), suggesting that the distance at which microtubules are maintained is critical to their sliding in the elongating spindle. The recently determined structure of the central portion of kinesin‐5 revealed a 4‐helix bundle comprising the antiparallel coiled‐coils of each kinesin‐5 dimer 85. The structure suggests that the 90° offset of the coiled‐coils at either end of the kinesin‐5 mini‐filament might enable the motor domains at either end of the filament to step processively along each microtubule, thereby permitting or constraining outward sliding within the mitotic spindle. The coiled‐coil assembly is proposed to be mechanically stable, capable of resisting tensile and compressive forces during spindle elongation.

Cytoplasmic dynein is a minus end‐directed motor protein complex that drives long‐range retrograde transport of cargo along microtubules. Dynein thereby plays a key role in cellular processes such as fast axonal transport, Golgi vesiculation, and mitosis. Crystallographic and cryo‐electron microscopy studies have provided important insights into the chemo‐mechanical cycle of dynein (reviewed in 68). The motor domain of dynein comprises a hexameric AAA+ ATPase from which emerges a 15 nm coiled‐coil stalk, at the tip of which is the microtubule‐binding domain (MTBD) (Fig. 4). Dyneins are conserved in both length and sequence, including the coiled‐coil stalk (Table 1). An α‐helical linker domain spans the AAA+ ring and is critical for force generation during the powerstroke 69, 70. The base of the stalk, emerging from the fourth AAA+ domain, is contacted by a second coiled‐coil called the buttress, from the fifth AAA+ domain. ATP binding in the motor domain is coupled with conformational changes in the stalk that are propagated to the MTBD, directly modulating its affinity for microtubules 71. Structural studies have provided direct evidence for the existence of two discrete registries of the coiled‐coil in the stalk 71, 72, 73, 74 (Fig. 4). Communication along the stalk involves the helices in the coiled‐coil sliding past each other by a full α‐helical turn, thereby shifting the position of a helix in the MTBD that sits at the interface with the microtubule (reviewed in 75). The trigger for helix sliding in the stalk was identified with the crystal structure of the ATP‐bound conformation of AAA1 69. Conformational changes in the AAA+ ring shift the buttress relative to the base of the stalk, pulling on one of the helices in the stalk and causing it to slide past the other (Fig. 4). This motion couples the motor domains to conformational changes in the MTBD that promote microtubule release. A recent study has proposed a modification of the helix‐sliding model, likening formation of the alternate coiled‐coil registries to an open zipper 76.

Kinesins, like dynein, transport cargo along microtubules, but toward the plus end. Kinesin‐1 comprises a N‐terminal “head” or “motor” domain with structural similarity to myosin 77, a C‐terminal “tail” involved in both cargo binding and autoinhibition 78, and a “stalk” domain in between that adopts a parallel coiled‐coil conformation that is extremely well conserved in length (Table 1). Kinesins function as dimers in which the “neck” coiled‐coil maintains the kinesin “heads” in close proximity 79. The neck linker, a 15 amino acid segment that joins the motor domain to the neck coiled‐coil and is structurally linked to ATP hydrolysis, is responsible for maintaining directionality during movement along microtubules 80. Kinesin‐1 elevates its cargo approximately 17 nm above the microtubule surface during transport 81, but the predicted length of the coiled‐coil stalk in an extended conformation is ∼60 nm. It is, therefore, clear that kinesin‐1 must exist in a more compact state. It is also clear that the stalk must contain regions of intrinsic flexibility in order to permit the autoinhibition of the motor domain by the C‐terminal tail 78. However, while the kinesin stalk is extremely well conserved in length and shortening of the stalk led to increased “limping” of kinesin in one study 82, cross‐linking of the coiled‐coil or mutations that increase stiffness appear not to make a difference to the frequency of limping 83. Future structural studies will be necessary to reveal the conformation(s) and regulatory function(s) of the stalk, both in the inactive and active states of kinesin‐1.

Kinesin‐5 motors are important for the assembly and elongation of the bipolar spindle in most eukaryotic cells. Unlike kinesin‐1, kinesin‐5 motors form a homotetrameric complex in which two pairs of motor domains are separated by a 79 nm, rigid coiled‐coil domain, conserved in length, but less conserved in sequence than kinesin‐1 (Table 1) 84. Displaying a preference for antiparallel microtubules, kinesin‐5 motors have been suggested to drive or constrain spindle elongation during anaphase. Recently, the structural basis for the assembly of the bipolar kinesin‐5 mini‐filament was revealed, showing how the antiparallel coiled‐coils of each motor complex pack together to form a 26 nm long four‐helix bundle at the center of the 79 nm rod 85 (Fig. 4). The high degree of length conservation in the coiled‐coil suggests that the arrangement and spacing of the motor domains at either end of the filament is critical to the sliding of microtubules in the growing spindle.

## Coiled‐coil domains function as molecular rulers

Enzymes increase the rate of a reaction by lowering the activation energy barrier. To do so, they must interact with their substrates in precisely defined ways. The presentation of substrate and/or enzyme is, therefore, a key regulator that controls the flux and fidelity of signaling in many pathways. In addition, the spatial and temporal dependencies of many processes necessitate the interaction of enzyme and substrate at specific subcellular locations. The compartmentalization of these signaling reactions is key to their switch‐like properties and the minimization of undesired crosstalk within the cell. Recent studies on diverse enzymatic processes from protein kinase signaling to O‐glycan chain synthesis have established the concept of a coiled‐coil “molecular ruler” – a mechanism by which enzymatic activity can be confined spatially with respect to its substrate.

**Figure 5:**
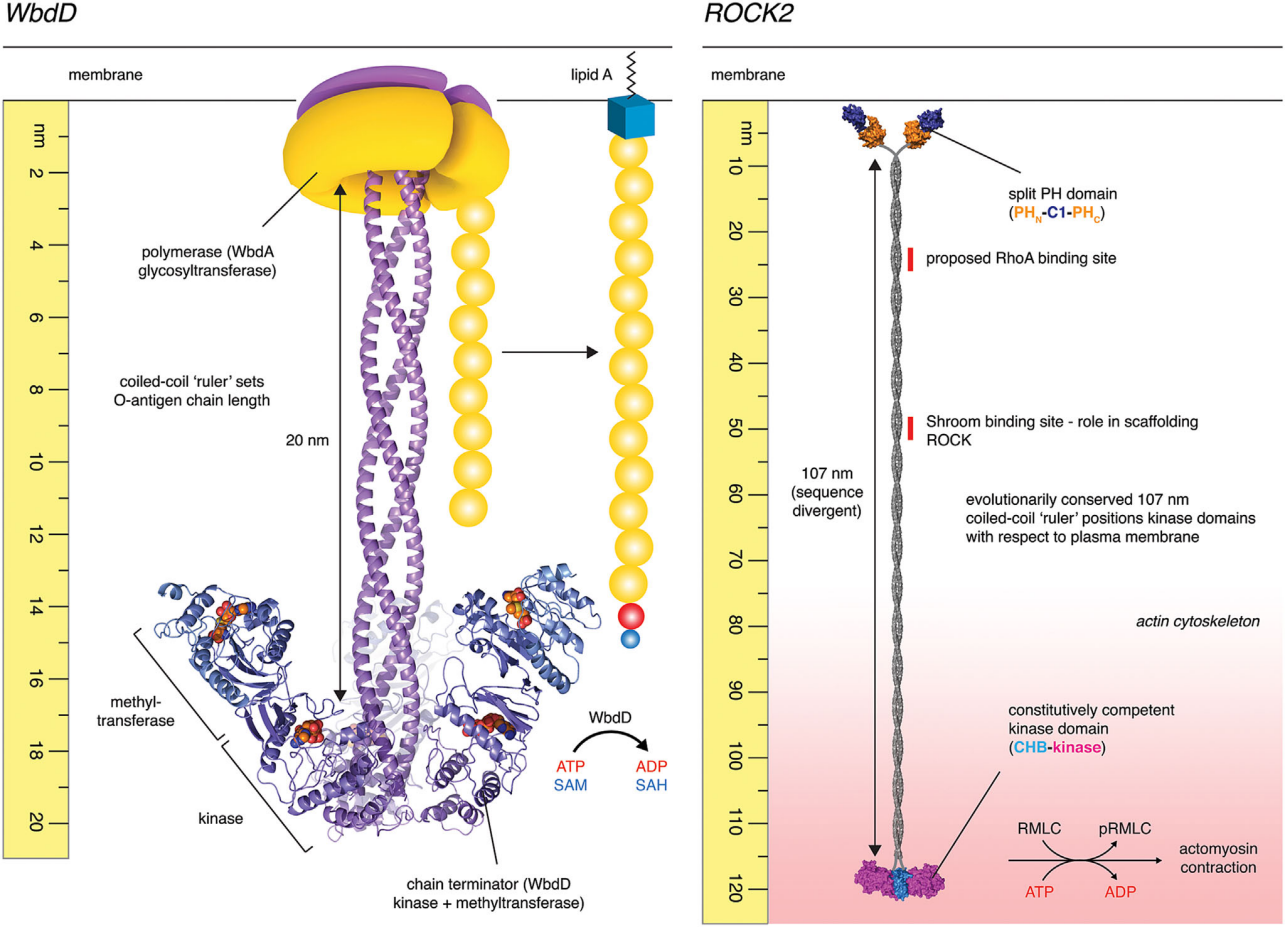
Molecular rulers. **WbdD.** O‐antigen synthesis in the pathogenic gram‐negative bacteria *E. coli* O9a is regulated by a polymerase, WbdA together with a capping enzyme, WbdD. Comprising N‐terminal methyltransferase and kinase domains followed by a C‐terminal coiled‐coil and transmembrane segment, WbdD assembles into trimers in which the catalytic domains are maintained at a distance of approximately 20 nm from the plasma membrane. Truncations in the coiled‐coil lead to a decrease in the modal length distribution of the O‐antigen polymannose chains 87. Source material for figure: G. Hagelueken and J.H. Naismith. **ROCK2.** Electron microscopy of full‐length human ROCK2 recently revealed it to be a constitutive dimer, 120 nm in length 90. N‐terminal kinase domains are separated from C‐terminal membrane‐binding domains by 107 nm of parallel coiled‐coil, which is evolutionarily conserved in length, but divergent in sequence (Table 1). ROCK2 is constitutively active in vitro and unaffected by membrane binding, RhoA, or activation loop phosphorylation. Truncations in the coiled‐coil, however, lead to defects in stress fiber formation in vivo, but do not affect catalytic activity. A new model proposes that the coiled‐coil of ROCK functions as a molecular ruler, positioning the kinase domains at a fixed distance from the plasma membrane, coincident with its substrates in the actin cytoskeleton.

Lipopolysaccharide (LPS) is a major component of the outer membrane of Gram‐negative bacteria, where it plays important roles in pathogenicity and viability. LPS comprises a conserved membrane anchor, lipid A, and a short conserved core oligosaccharide that links lipid A to an immunogenic polysaccharide, called the O‐antigen, of variable chain length. The O‐antigen has been shown to be vital for evading detection by the host immune system 86. While O‐antigen chain length is variable, it is notable that they fall in defined ranges that are O‐serotype specific. Hagelueken and co‐workers looked at how the O‐antigen is synthesized in the pathogenic bacterium, *Escherichia coli* O9a, in an effort to unravel the molecular basis for the defined chain length 87. In *E. coli* O9a, the O‐antigen is synthesized by a complex of two proteins, WbdA and WbdD (Fig. 5). WbdA is a polymerase, which adds mannose residues to the elongating chain, while WbdD terminates the chain by sequentially phosphorylating and then methylating the O3 position of the terminal mannose residue. WbdD comprises N‐terminal methyltransferase and kinase domains, followed by an extended coiled‐coil domain and a C‐terminal membrane interacting region. WbdD assembles into trimers, shaped like an umbrella, in which the catalytic domains are held at a fixed distance from the membrane by a three‐stranded parallel coiled‐coil (Fig. 5). Insertions or truncations in the coiled‐coil increase or decrease the modal chain length, respectively, suggesting that the function of the coiled‐coil is to maintain the chain‐terminating enzymatic activities at a fixed distance of ∼200 Å from the polymerase, WbdA, thereby ensuring a defined O‐antigen chain length.

A second example of a molecular ruler was recently found in the Rho‐associated coiled‐coil kinase (ROCK2), a serine/threonine protein kinase. The Rho kinases are critical regulators of the actin cytoskeleton, phosphorylating the regulatory myosin light chain (RMLC) to induce actomyosin contraction. Chemical inhibition of ROCK, or expression of a dominant negative kinase‐dead ROCK, results in the complete loss of actin stress fibers in cells 88, 89. Truebestein and co‐workers recently showed ROCK2 to be an extended particle in which dimeric kinase domains are separated from membrane‐binding regulatory domains by a long coiled‐coil of 107 nm 90. ROCK2 is constitutively active in vitro, unlike many protein kinases in which activity is dependent on phosphorylation, is allosterically regulated, or the kinase is maintained in an autoinhibited state by its regulatory domains. However, the coiled‐coil, while highly divergent in sequence between orthologs, has been conserved in length over approximately 650 m years of evolution (Table 1) 90, suggesting a functional significance. Truebestein et al. showed that, while truncation of the coiled‐coil had no effect on catalytic activity in vitro, expression of the truncated protein in cells resulted in a complete loss of actin stress fibers. The authors propose that the coiled‐coil may bridge the kinase domains of ROCK2 from the plasma membrane to the actin cortex and that ROCK2 activity is ultimately regulated by the spatial proximity of kinase and substrate (Fig. 5). In other words, a kinase can be constitutively competent without necessarily transferring phosphate to its substrate.

The implications of this model are profound. Only one kinase is widely believed to be constitutively active in the cell: casein kinase 2 (CK2) 91. The consequences of unregulated kinase activity are manifold. However, spurious phosphorylation could only occur in a system in which kinase and substrate(s) are free to interact. While the details regulating the spatial arrangement of ROCK2 and its substrates within this subcellular context remain a topic of investigation, regulating phosphotransfer could be achieved by changes in the position or conformation of either kinase or substrate. In the actin cortex, mechanical force transducers have been shown to adopt dramatically different conformations under different loads 92, 93, suggesting that the precise positioning of components in the cortex likely varies as a consequence of cytoskeletal tension. In terms of regulating ROCK positioning, the scaffold protein Shroom, which interacts with a small segment of the coiled‐coil of ROCK 94 (Fig. 5), has been shown to be essential for neural tube closure during embryogenesis 95, 96. In addition to a ROCK‐binding domain 97, Shroom is itself linked to the cytoskeleton by an actin‐binding domain, thereby providing a structural and perhaps mechanical bridge between kinase and substrates. Finally, it will be interesting to discover whether the ROCK family kinases, myotonic dystrophy‐related Cdc42‐binding kinase (MRCK), dystrophia myotonica protein kinase (DMPK), and citron Rho‐interacting kinase (CRIK) behave similarly. Both MRCK and DMPK contain long coiled‐coil domains, are conserved in length (Table 1), and phosphorylate the ROCK substrate RMLC.

## Less common than you might think – coiled‐coils as molecular scaffolds

Long coiled‐coil proteins encode an enormous repertoire of surface epitopes, in addition to potentially linking functional domains or communicating conformational changes. This raises the obvious question of whether in fact the surfaces of coiled‐coil proteins are widely used as molecular scaffolds. Surprisingly, examples of their use to scaffold multi‐subunit protein complexes or to recruit enzymatic activities 98, 99, 100, 101, 102, 103, 104, 105 are relatively scarce. Analogous to the interaction of Shroom with ROCK, the coiled‐coil domain of MRCK has been proposed to contain a binding site for the leucine repeat adaptor protein 35a (LRAP35a), which links MRCK to MYO18A in a tripartite complex responsible for the assembly of lamellar actomyosin bundles essential for cell protrusion 106. However, perhaps the best‐known example is tropomyosin, which stabilizes actin filaments and regulates actomyosin contraction. Tropomyosin is a two‐stranded parallel coiled‐coil approximately 40 nm in length, which binds to the actin filament in a head‐to‐tail arrangement, forming a pseudo‐continuous rope that twists around the length of the filament. Two studies have recently provided new insights into the molecular details of the interactions between tropomyosin, actin, and myosin 107, 108. While the conformation of tropomyosin is largely unaffected, the binding of myosin to the actin filament requires an azimuthal rotation of tropomyosin with respect to the filament axis. In this context, tropomyosin acts as a steric regulator of myosin binding; in skeletal muscle, elevated cytosolic calcium triggers conformational changes in troponin C, permitting the rotation of tropomyosin and initial myosin binding. This shift in tropomyosin exposes secondary myosin binding sites on the actin filament, leading to initial binding of the myosin head domain. Closure of the myosin head and binding to the actin filament is further stabilized by a direct interaction between myosin and tropomyosin 107.

The motor protein dynein depends on a large protein complex called dynactin for its function. Binding of dynactin and a cargo adapter converts dynein into a processive, unidirectional motor along microtubules 109, 110. The structures of dynactin in complex with dynein, with and without the cargo adaptor Bicaudal‐D2 (BICD2) were recently revealed by electron microscopy 111, 112. Both BICD2 and p150^GLUED^, a component of dynactin, are long coiled‐coil domain‐containing proteins. The structure of the dynactin‐BICD2 complex 111 reveals the structural basis for the requirement of the cargo adaptor for the stable interaction of dynein with dynactin. The 270 amino acid coiled‐coil domain of BICD2 extends along the length of the dynactin filament, providing a scaffold for the interaction of dynein and dynactin. This multivalent mode of interaction in the tripartite complex ensures that cargo is only picked up and transported by dynein in the presence of the adaptor.

Proteomic studies frequently pull out coiled‐coil proteins in screens for interaction partners, but most of the interactions remain to be validated biochemically and structurally. In this respect, it is worth noting that the interaction of RhoA with the coiled‐coil of ROCK has recently been challenged 90, 113. The majority of the evidence to date suggests that, while the concept of scaffolding by coiled‐coil proteins has been widely discussed, there are relatively few, structurally well characterized, examples of it in practice.

## Conclusions and future directions

The coiled‐coil is a remarkably versatile motif due to its inherent capacity to give rise to a polymer of, hypothetically, unlimited length and precisely adjustable mechanical properties. Coiled‐coil domains vary in length from the short to the extremely long, and from being almost invariant in sequence to hypervariable. Some coiled‐coils appear to function simply as molecular spacers while others have evolved the capacity to communicate conformational changes along their length. These properties make the coiled‐coil an essential component of the proteome, one that underlies the structure of organelles, facilitates critical maintenance, repair, and replication of genetic material, drives transport and trafficking of material within the cell, and correctly positions enzymatic activities.

While coiled‐coils represent a significant proportion of the eukaryotic proteome, there are relatively few high‐resolution structures of full‐length coiled‐coil‐containing proteins. Furthermore, crystal structures of coiled‐coils illustrate the plasticity of the motif, with many coils exhibiting non‐canonical packing interactions. Whether these structures, which are often fragments of the natural coiled‐coil, represent conformations along the normal trajectory is open to debate given the contributions of lattice packing interactions 114, and therefore interpretations of structural details, should be treated cautiously.

While the canonical coiled‐coil was postulated over 60 years ago, new twists on the possible structures that can be formed by coiled‐coils are still being discovered. Future challenges lie in understanding the mechanical properties of individual coiled‐coils and how they are suited for their biological functions. In this regard, the de novo design of novel coiled‐coil structures has the potential to enhance our understanding of the structure and properties of existing, naturally occurring coiled‐coils.
